# MRI of the Achilles tendon—A comprehensive pictorial review. Part one

**DOI:** 10.1016/j.ejro.2021.100342

**Published:** 2021-03-26

**Authors:** Pawel Szaro, Katarina Nilsson-Helander, Michael Carmont

**Affiliations:** a Department of Radiology, Institute of Clinical Sciences, Sahlgrenska Academy, University of Gothenburg, Gothenburg, Sweden; b Department of Musculoskeletal Radiology, Sahlgrenska University Hospital, Gothenburg, Sweden; c Department of Descriptive and Clinical Anatomy, Medical University of Warsaw, Warsaw, Poland; d Department of Orthopedics, Institute of Clinical Sciences, Sahlgrenska Academy, University of Gothenburg, Sweden; e The Department of Orthopaedic Surgery, Princess Royal Hospital, Shrewsbury & Telford Hospital NHS Trust, Shropshire, UK

**Keywords:** Achilles tendon, Spondyloarthropathy, MRI, Tendon, Achilles tendon xanthoma

## Abstract

The normal Achilles tendon is composed of twisted subtendons separated by thin high signal septae, which are a potential pitfall on MRI because they mimic a tendon tear.

Tendinopathy and full thickness tears may be assessed effectively both on MRI and ultrasound. MRI is superior to ultrasound in detection of partial tears and for postoperative assessment. The use of fat suppression sequences allows the ability to detect focal lesions. Sagittal and coronal sections are useful for assessing the distance between stumps of a ruptured tendon. Sequences with contrast are indicated in postoperative investigations and suspicion of infection, arthritis or tumor. MRI may reveal inflammatory changes with minor symptoms long before the clinical manifestations of seronegative spondyloarthropathy. The most common non-traumatic focal lesion of the Achilles tendon is Achilles tendon xanthoma, which is manifested by intermediate or slightly higher signal on T1- and T2-weighted images compared to that in the normal Achilles tendon. Other tumors of the Achilles tendon are very rare, whereas the involvement of the tendon from tumor in adjacent structures is more frequent.

The novel MRI sequences may help to detect disorders of the Achilles tendon more specifically before clinical manifestation. Regeneration or remodeling of the Achilles tendon can be non‐invasively detected and monitored in diffusion tensor imaging. Assessment of healing is possible using T2-mapping while evaluating the tendon vascularization in intravoxel incoherent motion MRI.

## Highlights

• Presence of normal septation between subtendons may mimic an intrasubstance tear.
• MRI is superior to ultrasound in detection of partial tears.
• Ultrasound is as useful as MRI in detection of tendinopathy and full-thickness tears.
• Kager's fat pad is involved in infection more than in postoperative changes.
• The Achilles tendon xanthoma has a higher signal on T1- and T2-weighted sequences.

## 1 The normal Achilles tendon

**Fig. 1:**
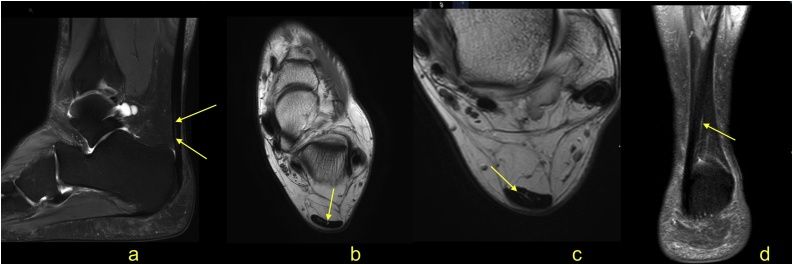
Normal Achilles tendon. A 32-year-old patient with clinical suspicion of a ganglion. MRI of the ankle (a-d) showed the higher signal within the Achilles tendon (arrows), which corresponds to the loose connective tissue between the subtendons. a - sagittal T2-weighted FS; b and c - axial section PD-weighted; d - coronal section PD FS.

The normal Achilles tendon is composed of three twisted subtendons; two from the medial and lateral heads of the gastrocnemius muscle and one from the soleus muscle [1,2]. The anterior outline of the Achilles tendon is mainly composed of a subtendon from the lateral head of the gastrocnemius. The medial part of the Achilles tendon consists of the soleus subtendon, while the posterior outline forms the subtendon from the medial head of the gastrocnemius [1,3]. The signal of the normal Achilles tendon on the PD- and T2-weighted sequence is low, with some thin high signal septae separating the individual subtendons (Fig. 1). The most consistently visualized septae are present in the anterior part of the tendon between the subtendons from the soleus and the lateral head of the gastrocnemius muscle. Normally present septation may be a pitfall on MRI and can be mistaken for a tear [[3], [4], [5]] (Fig. 1).

**Fig. 2:**
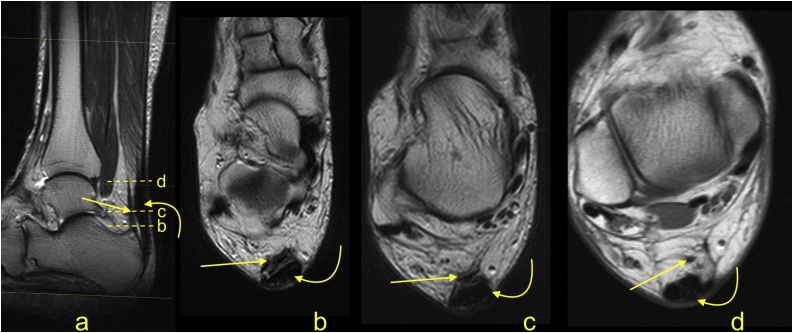
The plantaris tendon (straight arrow) contributes to the paratenon. A 48-year-old patient with pain in the Achilles tendon. Distally, the plantaris tendon (straight arrow) divides into small parts and merges with the paratenon, mimicking fibrosis on the axial section. The insertion is the calcaneus (straight arrow on figure a). The MRI (PD-weighted) revealed thickening of the Achilles tendon (curved arrow).

The Achilles tendon is covered by the paratenon, although normally it is not visible on MRI. The plantaris tendon contributes to the medial part of the paratenon in 40 % of patients [[6], [7], [8]] (Fig. 2). The paratenon is highly vascularized and thus is important in healing of the Achilles tendon [9]. Microscopically, the paratenon enters between the Achilles tendon fibers as the endotenon, where vessels, nerves, and tenocytes are located [10]. The enthesis of the Achilles tendon is a complex of the following structures accounting for the connection between the Achilles tendon and calcaneus; sesamoid cartilage and fibrocartilage, which are located at the anterior outline of the tendon; periosteal fibrocartilage covering the calcaneus; the retrocalcaneal bursa; and the part of Kager's fat pad that protrudes into the bursa [[11], [12], [13]]. The retrocalcaneal bursa is a synovial lined structure occupying the space between the Achilles tendon and calcaneus. The superficial calcaneal bursa is located between skin and the Achilles tendon insertion [1].

## 2 MRI vs. ultrasound in the assessment of the Achilles tendon

Ultrasonography is the most commonly used imaging modality for the assessment of the injured or painful tendon. The indications for MRI and ultrasound of the Achilles tendon somewhat overlap; however, a clinical examination is significant (Table 1) [14]. Ultrasound is a cheap and easily accessible modality; however, it is operator dependent, whereas MRI depends on an optimal protocol. Moreover, a different selection of patients for the previous studies makes an unambiguous comparison difficult. At our institution, postoperative changes are assessed by MRI with contrast because of the diversity of possible complications (Table 1). Clinical suspicion of arthritis, infection or tumor should be imaged with IV (intravenous injection) contrast. The degree of bursitis and presence of a tendon lesion within the insertion may also be determined effectively on MRI. Postoperative complications, such as calcaneus fracture, infection and abscess formation, tendon or graft rupture, can be assessed on MRI with contrast. Ultrasound is as good as MRI in detection of tendinopathy and full-thickness tears [15,16]. However clinical findings in tendinopathy correlate more with MRI than with ultrasound [17]. MRI is superior to ultrasound in detection of partial tears [16]. The use of new MRI sequences enables the early identification and differentiation between the types of tendinopathy, which is not possible in ultrasound at all [16]. In the enthesopathies, the degree of bursitis, the presence of tendon changes, and the presence of bone marrow edema in the bony insertion can be determined on MRI. Ultrasound allows dynamic assessment of the Achilles tendon, which is not possible on static MRI unless dynamic sequences are used.

**Table 1:** Use of MRI and ultrasound for diagnosing the most common conditions of the Achilles tendon diseases at our institution.

Condition	MRI	Ultrasound	Comments
Paratendonitis	++	+	Peritendinous fatty tissue may be assessed more reliable. Paratendonitis may be underdiagnosed [21].
Partial rupture	++	+	MRI is superior in the diagnosis of partial thickness tear [16].
Total rupture	+	++	Both MRI and ultrasound are appropriate diagnostic methods [16].
Midportion tendinopathy	+	++	Ultrasound and MRI are reliable; however, midportion tendinopathy may be overestimated in ultrasound [21,22].
Midportion tendinopathy with a partial tear	++	+	MRI is superior in differentiating pure tendinopathy from tendinopathy with partial ruptures [16,22].
Insertional tendinopathy	++	++	Ultrasound and MRI play complementary roles [23,24].
Acute postoperative changes	++	+	MRI and ultrasound may be used [25,26]. However, in each case, the assessment should be individual as it is a very heterogeneous group of patients.
Monitoring effects following different treatment	++	+	Monitoring of postoperative changes and therapeutic outcomes of spondylarthritis [11,20,22].
Tendon elongation	+	++	Easier simultaneous comparison with the healthy side [27].
Infection	++	+	MRI shows soft tissue and bone involvement [28].
Entesitis in spondylarthritis and psoriatic arthritis	++	+	MRI showed bone marrow edema, which correlates with the level of HLA-B27 [19].
Tumors and tumor-like lesions	++	–	Better differentiation of malignant and benign changes.
Imaging guided treatment	–	+	Easily localization of the needle in real-time. An easy technique for local injection therapies drainage of abscess and biopsy [29].
Dynamic assessment	–	+	Ultrasound allows easy dynamic assessment [25,30].

MRI – magnetic resonance imaging. Symbols used in the table: ++ the preferred method, + the alternative method, - the method is not used.

Hypervascularization in the Achilles tendon can be assessed by Doppler ultrasound, in MRI novel sequences or in classical MRI IV contrast.

Ultrasound elastography is used to distinguish the normal tendon from the pathologic tendon, allowing earlier diagnosis and monitoring of remodeling and treatment. However, more studies are required to evaluate the clinical value of this method. MR elastography is a new method used mostly in research (discussed in the section regarding new MRI techniques).

Both ultrasound and MRI are used to assess heel involvement in spondyloarthritis. Erosions at the enthesis may help to differentiate peripheral spondyloarthritis from non-inflammatory lesions [18]. Neither MRI nor ultrasound could differentiate between inflammatory lesions without erosions [18]. However, only MRI showed bone marrow edema, which correlates with the level of HLA-B27 [19]. Thus, differentiation from mechanically induced disease may be performed in doubtful cases. Sequences with fat suppression and contrast enhancement are appropriate methods of visualizing active enthesitis [20].

## 3 Protocol

A MR protocol for the Achilles tendon should include sequences in all three orthogonal planes. Fat suppressed imaging with T2- or PD-weighted images and at least one T1-weighted sequence for bone marrow evaluation are recommended (Table 2). Using fat suppression allows to increase the contrast between the free water and collagen fibers.

**Table 2:** MRI protocols used at our institution for assessment of the Achilles tendon on 3 T machine. Field of view is anterior-posterior 16 cm, right-left 11 cm and cranio-caudal 20 cm.

		TE [ms]	Range of TR [ms]	Time of scanning [minutes: seconds]
Protocol without contrast	T1 SE tra	10	450−750	3:31
	PD TSE SPAIR tra	45	2500−5000	2:24
	PD TSE sag	30	2000−5000	1:58
	T1 SE sag	11.5	450−750	1:42
	T2 TSE SPAIR cor	60	3000−5000	3:18
	STIR TSE sag	60	2500−3000	2:23
Protocol with contrast	T1 SPAIR TSE tra	9.5	450−750	4:09
	T2 TSE tra	80	3000−5000	3:17
	T1 TSE FS tra Gd	9.5	450−750	3:11
	T1 TSE FS sag Gd	10	450−750	1:20
	T1 TSE FS cor Gd	10	450−750	1:09

Abbreviations used in the table in alphabetic order. Cor - coronal section, FS - fat suppression, Gd - gadolinium, PD - proton density, SE - spin echo, SPAIR- Spectral Attenuated Inversion Recovery, TE - echo time, TR - repetition time, TSE - turbo spin echo, sag - sagittal section, STIR - Short-TI Inversion Recovery, tra - transverse section.

Sagittal and axial sections are the most useful in evaluation of the Achilles tendon. T2-weighted images are useful due to their high ability to demonstrate the fluid accompanying tendon pathology. The protocols used most often at our institution are provided in Table 2. Sequences with contrast are used in postoperative investigations and suspicion of infection, arthritis or tumor. In other cases, we used protocols without contrast (Table 2).

## 4 New MRI techniques

Evaluation of the microscopic properties of the Achilles tendon with conventional MRI sequences is limited because of a very short T2- time. The application of new MRI sequences may help to identify subclinical lesions, enabling to us to differentiate between different sorts of tendinopathies. The novel techniques are based on the alteration of the normal collagen fibers by water, which change local homogenic structures and thus magnetic features of the Achilles tendon.

Modification of classical diffusion tensor imaging (DTI) MRI may reveal tendinopathy-induced microstructural disorganization in the very early stages. The process of regeneration, healing, remodeling and progress or treatment response are possible applications of DTI [31]. Assessment of the microarchitecture of the Achilles tendon after reconstruction with the possibility of the fiber tracing is a possible application of DTI [26,32].

T2-mapping is a promising method to assess tendon healing. The normal tendon has a shorter T2 value compared to that in the injured part of a tendon [33]. The concentration of water is higher after trauma and then decreases with the time, thus the healing process may be quantified using T2-mapping [34]. It may be used clinically as robust biomarker of tendon healing, making for easier decisions regarding the appropriate time to return to sport [33].

Low vascularization of the Achilles tendon may complicate the process of healing. The development of vascular networks after injury may be crucial for Achilles tendon healing. The microcirculation and microvasculature properties of the Achilles tendon could be assessed using an intravoxel incoherent motion MRI [35].

MR spectroscopy is a promising method for evaluating metabolic activities and biochemical composition. Detecting different signals from different molecules may help to assess the level of water, inorganic phosphate or adenosine triphosphate [36]. This method is used mostly for muscles, and application in tendons is technically challenging because of short T2 time. Ultrashort sequences allow quantitative imaging of water within the Achilles tendon. Direct imaging of water within the tendon may be a favorable way of diagnosing tendon disorders [37].

Pathological tendons exhibit higher levels of glycosaminoglycans, which attract sodium ions. Sodium MRI may help to evaluate the level of sodium in the tissue, allowing indirect evaluation of glycosaminoglycan content within the tendon [38]. Sodium MRI may reveal changes in the Achilles tendon during treatment with ciprofloxacin, while other new sequences, even T2*, show neither tendon lesions nor clinical symptoms are present [39]. Thus, sodium MRI may be applied in the prevention of tendon rupture in asymptomatic patients treated with fluoroquinolones.

MR elastography is used to assess the mechanical properties of a muscle, mainly in neuromuscular disorders [40], but clinical application to tendons has not been done yet [32].

## 5 Infections

**Fig. 3:**
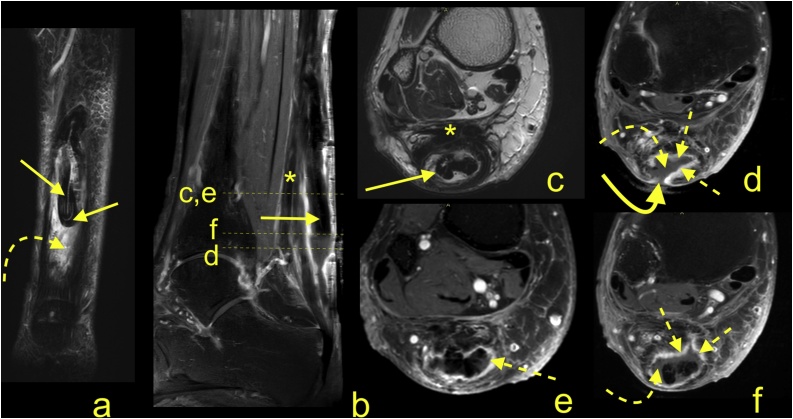
Rupture and infection of the Achilles tendon graft. A 76-year-old patient received a tendon graft because of a chronic Achilles tendon rupture 6 months ago. She had a wound that would not heal, despite treatment with negative pressure wound therapy. Fever and malaise in the past few days and worse function of the reconstructed tendon. MRI (a-f) with contrast was performed. Rupture of the graft with retraction was revealed (straight arrow). Fluid collection with enhanced walls (dashed arrows) around the graft and fistula (curved arrow) is visible. Extensive thickening of the paratenon (*) and adhesions in Kager's fat pad are present. A- T2- weighted with fat suppression, b- T1- weighted with fat suppression and contrast, c- T1- weighted, d, e and f - T1- weighted with fat suppression and contrast.

**Fig. 4:**
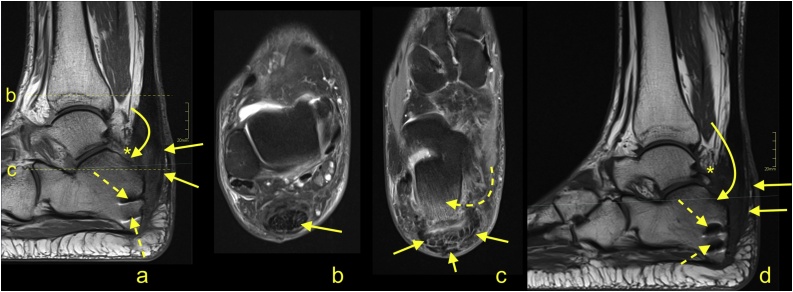
Postoperative infection of the Achilles tendon insertion after surgery for Haglund's deformity. Surgery was performed 5 weeks ago. The wound is not healing, and now, there is redness and swelling of the Achilles tendon insertion. Alteration of the structure, loss of fibrillar structure of the tendon with edema, and reactive changes in the subcutaneous tissue are visible (straight arrow); however, it is challenging to distinguish postoperative changes from infections based only on MRI (a-d). The presence of abscess was ruled out after contrast injection (not seen in the figure). A postoperative defect in the calcaneus (curved arrow) and adhesions with alteration in Kager's fat pad are visible. Diffuse bone marrow edema in the calcaneus is present. Screws in the calcaneus (dashed arrow). a- PD-weighted; b and c - PD-weighted with fat suppression; d - T1-weighted.

Infection of the Achilles tendon and paratenon are rare and often are complications of Achilles tendon repair or reconstruction (Fig. 3), Haglund debridement (Fig. 4), penetrating or non-healed wounds or open lacerations [25,41]. Infection may be difficult to distinguish from postoperative changes, especially in the early stages; however, infection usually causes more extensive and diffuse reactive changes within Kager's fat pad. Administration of IV contrast allows the exclusion of abscess formation (Fig. 3, Fig. 4) [42].

**Fig. 5:**
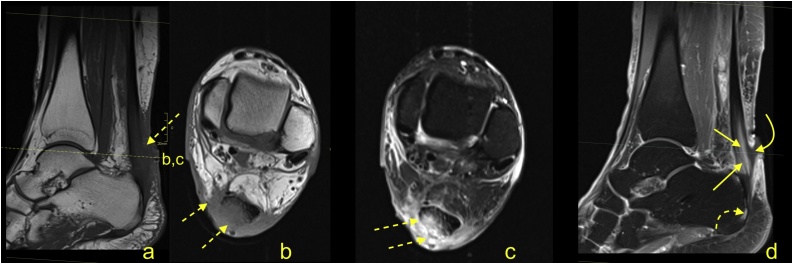
Infection of the Achilles tendon because of a non-healing wound on the heel in a 68-year-old patient. MRI (a-d) revealed extensive edema of the skin and subcutaneous tissue (dashed arrow) around the fistula (curved arrow). After contrast administration, enhancement is visible in the Achilles tendon (straight arrow) at the fistula level. A small area of bone marrow edema in the calcaneus (curved dashed arrow) is probably not related to inflammation. A- T1- weighted, b- T1- weighted with contrast, c and d- T1- weighted with fat suppression and contrast.

Wounds and surgical incisions in the Achilles tendon area are more prone to infection and wound breakdown compared with other areas of the body because of worse vascularity (Fig. 5). This may be revealed as a thickened tendon and paratenon. Reactive changes are visualized as edema in the subcutaneous tissue and in Kager's fat pad. Following the administration of contrast, diffuse enhancement in the Achilles tendon, the paratenon and Kager's fat pad is seen (Fig. 3). A fistula may form between the skin and an abscess collection, particularly on the posterior aspect of the tendon (Fig. 5).

## 6 Arthritis

### 6.1 The Achilles tendon in seronegative arthropathies

**Fig. 6:**
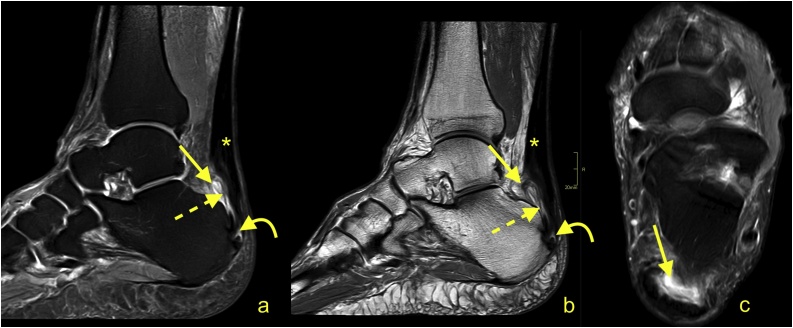
A 26-year-old patient with a suspicion of spondylarthritis presenting with discomfort in the insertion of the Achilles tendon. a and c- T2-weighted with fat suppression; b - T2-weighted. MRI revealed retrocalcaneal bursitis (dashed arrow) and bone marrow edema in the Achilles tendon insertion, however no erosions are present. Thickening of the Achilles tendon in midportion is visible (*), however no alteration in the signal of the Achilles tendon was revealed. A-T2- weighted, b- PD- weighted, c- PD-weighted with fat suppression.

Enthesitis is a key sign of spondylarthritis and psoriatic arthritis [11,43]. The perientheseal inflammation may be seen in early peripheral spondylarthritis and can be revealed on MRI as bone marrow edema in the calcaneus, allowing detection of subclinical cases (Fig. 6). The higher signal in the Achilles tendon and subcortical bone marrow edema at the insertion are MRI features and correlate with higher levels of HLA-B27 [19]. Erosions are present in one-third to two-thirds of patients and may help differentiate peripheral spondyloarthritis from other cases [18,44]. Tendon thickening, retrocalcaneal bursitis, edema of Kager's fat pad, erosions, bony irregularities, and enthesophytes of the plantar fascia may be seen on MRI in patients with spondyloarthritis. Hypervascularization of the enthesis may be easily evaluated by Power Doppler examination [45]. For visualization of active enthesitis, MRI sequences with contrast are recommended [20]. Assessing the degree of enthesitis is essential to evaluate disease activity and to monitor treatment [11,46].

Enthesopathies are pathological changes at the tendon insertion and can also be seen in degeneration, metabolic syndromes, endocrine disorders, and traumatic or mechanical conditions [11].

### 6.2 The Achilles tendon in rheumatoid arthritis

Enthesitis is not a hallmark of rheumatoid arthritis; however, some patients have an altered signal within the midsubstance of the tendon but without the thickening associated with classical tendinopathy [47]. The retrocalcaneal bursa may also have a higher signal, although there may be a paucity of clinical features [47]. Conversely, some patients have tendon thickening without a typical focal intratendinous signal. Early retrocalcaneal bursitis may be seen because inflammation ascends from the synovial membrane lining the walls of the bursa [48].

## 7 Tumors

Tumors are uncommon in the Achilles tendon and "tumor-like" lesions occur more frequently. If a tumor is present, it is often benign. The differential diagnosis of a mass on the Achilles tendon is t tendinopathy, along with lesions such as xanthomas, giant cell tumors or fibromas. Loss of the anterior concavity of the Achilles tendon, a painless soft tissue mass occurring most commonly bilaterally and symmetrical at the distal portion of the Achilles tendon may be seen in Achilles tendon xanthoma [49]. The accumulation of lipid-laden macrophages within the Achilles tendon results in an intermediate or slightly higher signal on T1- and T2-weighted images compared to those in the normal Achilles tendon [50].

**Fig. 7:**
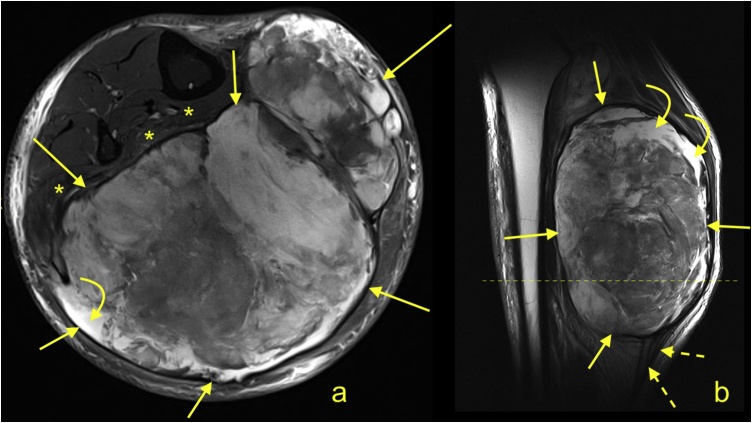
Large soft tissue tumor in the triceps surae muscle (histology showed undifferentiated pleomorphic sarcoma). A 43-year-old patient under treatment for the tumor. The control MRI showed necrosis in the tumor (curved arrow). The aponeuroses of the gastrocnemius and soleus muscles, as well as the Achilles tendon, are dislocated (dashed arrow) because of mass effect. a - PD-weighted; b - T2-weighted.

A giant cell tumor is an extra-articular form of pigmented villonodular synovitis. Additionally, diffuse-type tenosynovial giant cell tumors have been reported [51]. MRI reveals an intermediate to low signal on T1- and T2- weighted images.A fibroma of the Achilles tendon is frequently a well-circumscribed mass and most commonly observed in children. MRI demonstrates a lower signal on T1- and T2-weighted images. If contrast is administered, mild heterogeneous enhancement may be seen.If malignant lesions involve the Achilles tendon, it usually occurs by local infiltration or mass effect from the adjacent tumor (e.g., originating from the gastrocnemius) (Fig. 7).

**Fig. 8:**
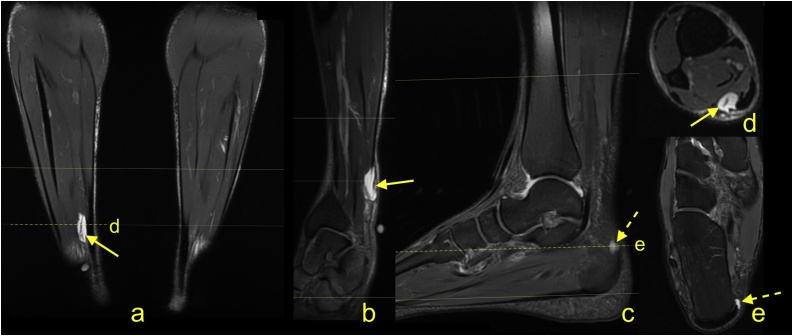
A 27-year-old athlete presents with a palpable focal thickening of the medial part of the Achilles tendon. Clinical suspicion of lipoma. MRI revealed a ganglion-like structure on the medial part of the midportion of the Achilles tendon (straight arrow). A similar lesion is visible on the medial side of the insertion (dashed arrow). No contrast enhancement was noticed, and the radiological diagnosis was ganglions. Surgery was performed, and the proximal lesion was removed. The postoperative diagnosis was ganglion. a-e - PD-weighted with fat suppression.

**Fig. 9:**
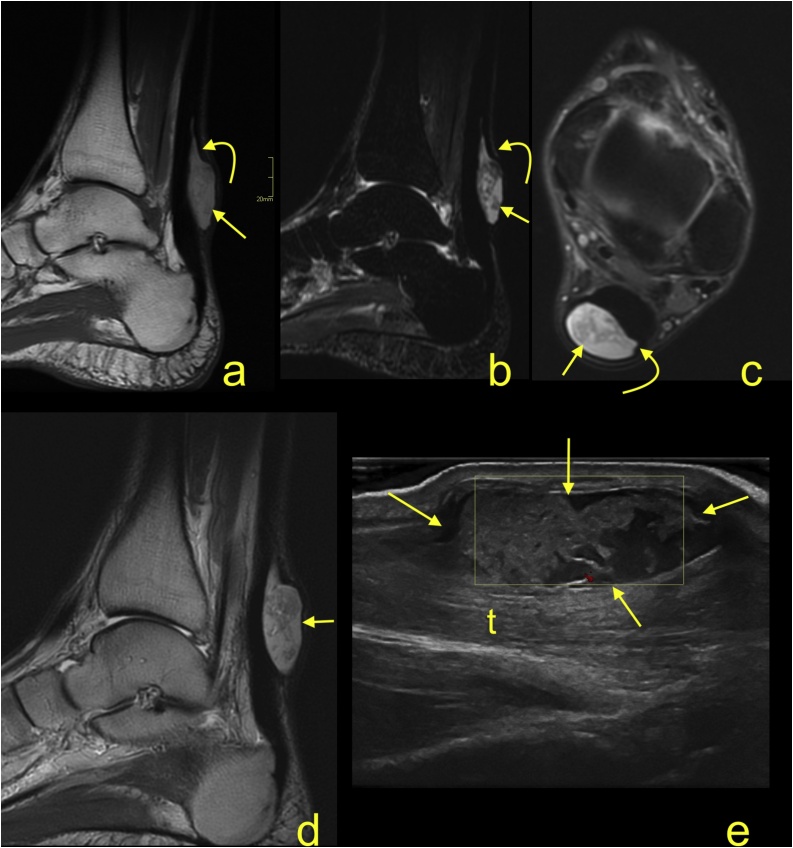
A 33-year-old patient who presents after suffering a direct injury to the Achilles tendon. There was neither rupture nor tendon dysfunction revealed on clinical examination. The posttraumatic focal swelling was understood as a hematoma. After 2 weeks, when the clinically suspected hematoma did not decrease in size, an MRI was performed. The heterogeneous fluid lesion with a higher amount of protein (a) (straight arrow) is located between the paratenon (curved arrow) and Achilles tendon. a - T1-weighted; b - STIR; c - PD-weighted with fat suppression; d - T2-weighted. An ultrasound-guided puncture was performed, and clear serous fluid was aspirated. e - ultrasound correlation is presented (longitudinal sagittal section, t - Achilles tendon).

Most tumor-like focal lesions within the Achilles tendon are related to injury and are fluid-filled intrasubstance tears. In rare cases, a ganglion-like lesion may be found with typical features (Fig. 9). Low-protein ganglions give a high signal on T2-weighted images and a low signal on T1-weighted images without contrast enhancement. Sometimes, multiple ganglia may be present (Fig. 8). Direct trauma to the Achilles tendon may result in a hematoma or seroma located on the tendon's surface at the site of direct injury (Fig. 9).

## 8 Summary

The indications for MRI and ultrasound overlap, and there is no clear consensus for the use of imaging diagnostics in the diagnostic of the Achilles tendon. Appropriate MRI protocols and novel MRI sequences may help to differentiate disease entities from similar clinical manifestations. The novel sequences are the future of Achilles tendon imaging because they enable treatment monitoring and early detection of tendinopathy. In infection, it allows assessing the involvement of bone in osteomyelitis. Administration of contrast enables the exclusion of abscess. The higher signal in the Achilles tendon and subcortical bone marrow edema at the insertion are MRI features of seronegative arthropathies. Achilles enthesitis revealed on MRI precedes the onset of systemic disease. MRI helps to differentiate true tumors from tumor-like lesions.

## Funding

This project did not receive any specific grant from funding agencies in the public, commercial, or not-for-profit sectors.

## CRediT authorship contribution statement

**Pawel Szaro:** Conceptualization, Investigation, Visualization, Supervision, Writing - original draft, Investigation, Writing - review & editing. **Katarina Nilsson-Helander:** Writing - review & editing. **Michael Carmont:** Writing - review & editing.

## Declaration of Competing Interest

The Swedish Ethics Committee approved the study and waived the need for informed consent (2020-06177). This project did not receive any specific grant from funding agencies in the public, commercial, or not-for-profit sectors. The authors declare that there is no conflict of interest.
